# Does Health Promotion Program Affect Local Resident’ Emotions?

**DOI:** 10.3390/ijerph16040549

**Published:** 2019-02-14

**Authors:** Munjae Lee, Sewon Park, Kichan Yoon

**Affiliations:** 1 Department of Medical Device Management and Research, SAIHST, Sungkyunkwan University, Seoul 06351, Korea; emunjae@skku.edu (M.L.); se10919@g.skku.edu (S.P.); 2 Research Center, Social Security Information Service, Seoul 04554, Korea

**Keywords:** healthy city, health promotion, local resident emotion, happiness, Korea

## Abstract

Healthy cities continuously attempt to improve residents’ health. Health is affected by psychological factors, such as happiness and emotions. Therefore, this study investigates the effects of healthy city program performance on individuals’ emotions, as well as the correlation between healthy city program performance and emotions using personal happiness index as a parameter. We conducted a questionnaire survey of residents in areas implementing healthy city projects. A total of 596 responses were obtained. We used structural equations to analyze the relationship of structural influences. Results showed that healthy city program performance had significant static effects on emotion. This observation shows that healthy city programs decrease local residents’ negative emotions, such as stress and depression. Therefore, healthy city programs stabilize residents’ emotions by increasing health friendliness. To improve the performance of healthy city programs, it is necessary to mitigate health risk factors and positively affect individuals’ emotions.

## 1. Introduction

The healthy city paradigm was introduced in the 21st century to improve mortality and quality of life based on New Public Health. The idea of healthy cities was introduced by the World Health Organization (WHO) in 1984 to promote healthy cities and communities; Ottawa was selected as the target of the first healthy cities project [1]. Healthy cities maximize the use of resources during physical and social development to allow for expression of members’ full capacities. Furthermore, healthy cities continuously seek to improve residents’ health through cooperation. In other words, healthy cities attempt to improve social settings using the creativity of local government and society. In Korea, the first attempt to develop a healthy city was begun in 1996 in Gwacheon, Gyeonggi-do. In 2006, the Korean Healthy Cities Partnership was founded to sponsor national healthy city programs, share data, and promote health equality. As of 2018, 93 full member cities and 11 associate member cities actively promote healthy cities as members of the organization [2,3].

A healthy city program links a city’s living conditions with the citizens’ health and considers health promotion as a basic unit of the community. Healthy cities should lead local communities to participate in local governments and strive to improve the cities’ health and structure. A healthy city program is conducted by a local government based on the assumption that the local government’s interest in the health of the local residents actually improves the health status of the residents.

Healthy city programs include healthy urban environment projects, healthy living space projects, healthy living practices, and health services. Healthy urban environment projects include ecological river restoration, fine dust concentration improvement, active bicycle use, water quality inspection visit services, and healthy town developments. Healthy living space projects include maternal and child health programs, youth health promotion programs, elderly welfare projects, healthy home-building projects, multicultural family visiting education, school meal services, child food safety management, healthy and safe school management, and disease-free longevity classes. There are alcohol prevention education programs, smoking cessation clinics, revitalization of life sports, and child health experience classrooms as healthy life practices. The programs that strengthen health services include Cardiopulmonary Resuscitation (CPR) training, prevention and management of atopic diseases, internal correction projects, mental health programs, vaccination businesses, and suicide prevention projects.

A healthy city program is a project operated by individual local governments. Therefore, it is mainly operated by the health promotion departments in welfare centers and healthy city teams within the local governments. As part of their initiatives, these programs encourage the participation of all residents of an area in conducting projects to build healthy cities. As a result, healthy city programs can be found in living environments in the community, such as in medical and sports facilities.

Evaluation of healthy cities is necessary to determine whether the activities of local governments have achieved significant improvements in city health. The optimal state of a healthy city includes public policy-making with the participation of local society members. To induce long-term changes in local society, a healthy environment must be established and society’s ability to solve health-related problems should be continuously improved. The process of development into a healthy city is determined crucially by the local government’s leadership and will to change. New policies, programs, services, or resources under local leadership change the city’s environment and can improve the level of health in local society. Evaluation is also necessary to promote participation and cooperation of society members and to establish a rationale for promoting the healthy city program [4,5].

Happiness is a variable that expresses a positive mindset and includes both physical and psychological health. Therefore, health and happiness are correlated. The human body perceives happiness as good health, which further reduces pathological symptoms and risk of disease. Psychological variables play a more important role than external variables on health, with internal variables greatly affecting subjective health levels. Subjective welfare, which refers to self-evaluation of one’s satisfaction with life and emotional state, has a strong causal correlation with health and mortality. When individuals experience depressed or joyful moods, they perceive a decrease or increase, respectively, in their quality of life [6,7,8]. Because health is an important factor for happiness, a healthy city’s environment can affect both health and happiness. As health deteriorates, happiness decreases. Similarly, as expected income from society increases, happiness increases. Therefore, the performance of healthy cities programs are expected to vary from city to city [9]. Social capital creates the infrastructure for social cohesion. Social cohesion refers to the characteristics of a society that include strong solidarity, conflict management systems, and no social conflicts, such as inequality, discrimination, and polarization. Social capital can be associated with depression, violence, and health status. Health can be improved when both individual and collective efforts are harmonized. Social capital has a high impact on health promotion, which is observed as a capacity measure of a community. Therefore, a healthy city program for solving health inequality is considered to increase the social capital of the local residents. In addition, the development of conducive physical environments, such as walking paths and promenades, in the facilitation of healthy cities appears to be an improvement in the practice of healthy living. Considering the characteristics of social capital, it is possible to achieve more effective healthy city program performance by strengthening the capacity of local residents and their participation in community healthy city programs [10].

Individual emotional state and physical health in daily activities are known to be closely correlated. Individuals with high emotional perception are less affected by stress than individuals with lower emotional perception. Furthermore, they are protected from negative emotions that can lead to depression and suicidal ideation [11]. Depression levels increase as life satisfaction decreases; life satisfaction decreases with increases in stress. Disease, a known life stressor, causes high levels of depression. However, anxiety is more common than depression as a cause of stress. Individuals with high anxiety are more predisposed to develop depression with increases in their stress level; optimistic individuals have an outstanding ability to deal with stress in circumstances of high negative emotion, such as depression and anxiety. Improper life habits, such as smoking and alcohol dependence, also increase stress levels, which leads to unstable emotions and ultimately to disease. The ability to deal with such stressors varies based on an individual’s emotional stability [12].

Health is directly affected by psychological factors, such as happiness and emotions. As healthy city program performance improves, the emotional stability of local residents is expected to increase. The present study investigated the effects of healthy city program performance on individuals’ emotions and the correlation between healthy city program performance and emotions using personal happiness index as a parameter. The results could indicate the ideal direction for healthy city programs to follow and important variables for further improving program performance.

## 2. Materials and Methods

**Figure 1:**
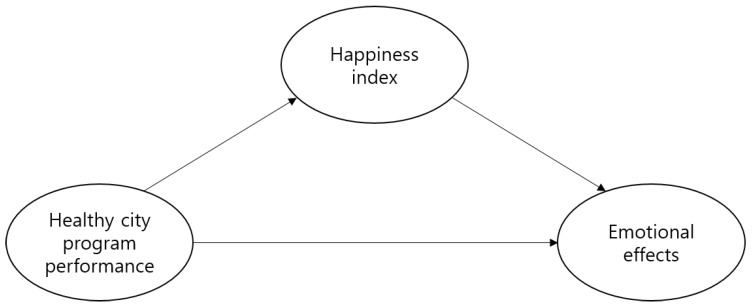
Research model.

This study’s objective was to qualitatively define the correlation between a healthy city program’s performance and the local residents’ emotions using personal happiness index as a parameter. The healthy city program performance was used as an independent variable and emotional effects were used as dependent variables. Personal happiness index was defined as a parameter between healthy city program performance and emotions. As previously discussed, psychological variables are closely correlated to health, and the happiness index, which reflects individual psychological state, ultimately affects the emotions. Therefore, the study design assumed that the healthy city program’s ultimate goal was improvement in local residents’ psychological health. The research model is illustrated in Figure 1 and details of the study design are described below (IRB code: 1040548 - KU-IRB-17-125-A-2). 

### 2.1. Definition of the Variables

#### 2.1.1. Healthy City Program Performance

Healthy city programs seek to enhance individual health through social, cultural, and economic development. The concept of the healthy city program is based on health benefits obtained from one’s environment. Because healthy city programs require the participation of personnel from different areas, the evaluation process is complex. Duhl (1992) [13] suggested that the program’s health promotion strategy should be included in evaluation, along with assessment of residents’ health. For further development of existing healthy city programs, evaluation focusing on the program’s initial stage is necessary [14]. Healthy city programs aim to measure the local community’s health status and to improve the community’s quality of life. Public policies aimed at improving local residents’ physical activity result in a benefit to public welfare. Additionally, improvement in community health through healthy city programs improves cohesion, social capacity, and social resources, leading to positive change. Therefore, many attempts have been made to evaluate the process and structure of city health improvement. In European healthy cities, an attempt has been made to establish not only a local but a national framework. The WHO’s European regional office announced a minimum recommended basic strategy, infrastructure, performance, and networking protocol. The Alliance for Healthy Cities (AFHC) has developed the SPIRIT checklist for evaluating healthy city programs, which incorporates accessibility, sustainability, political beliefs, policy, local community participation, data/innovation, research, infrastructure, and ability enhancement. These parameters are used to evaluate outstanding healthy cities. However, although attempts are made to evaluate healthy cities using demographics, politics, economy, and social issues, a detailed method for assessing local residents has not been suggested [15,16,17].

#### 2.1.2. Effects of Emotion

Health promotion refers to active, positive participation of individuals in disease treatment and management to improve or maintain their health. Not only does this improve physical health, it also has positive effects on psychological health, such as decreased depression [18]. Emotions are shown when individuals are aware of and appropriately cope with changes in the surrounding environment. Emotional stability refers to one’s adaptation to life circumstances with a positive attitude and a healthy psychological state. When emotions deviate from a specific range or experience extreme changes, emotional instability arises. Emotional instability plays an important role in understanding depression and its characteristic symptoms [19]. Depression affects the body by increasing its susceptibility to disease; conversely, bad health can also increase emotional hardship [20].

Health promotion that protects the health status and prevents the occurrence of health problems in an individual’s daily life can contribute to the emotional stability of human beings. The practice of health promotion positively affects emotional influences such as quality of life, psychological well-being, as well as physical health. Performing certain activities for improvement of health can provide a sense of security to local residents’ emotions. Some of the health promotion programs in the healthy city programs include drug addiction treatment, drinking and smoking cessation, and urban development for citizens’ emotional stability. In this way, a healthy city program has a positive effect on the emotions of the local residents because it eliminates negative physical and mental factors.

#### 2.1.3. Personal Happiness Index

Happiness is known to be correlated with quality of life, life satisfaction, and subjective stability. An individual who subjectively feels positive emotions due to appropriate experiences is defined as happy [21]. Happiness is a personal and subjective element of well-being. Happiness and health are also mutually influential factors. Healthy cities can directly affect the abstract concepts of the quality of human life and happiness in our lives. A happy person perceives themselves as being healthier, and those with a higher need for health view happiness as being more aware. Therefore, in this study, happiness was regarded as a personal emotion and is included in the personal happiness index [1]. The happiness index is a value that represents overall happiness by quantifying daily life activities, social value, and quality of life. This indicator of happiness is used to evaluate local policy-making [22]. It measures health-affecting variables such as psychological health, physical health, work/life balance, education, social relationships, the natural environment, local social policies, and social welfare. Community happiness can potentially facilitate continuous development of local societies [23].

### 2.2. Tools

Edmundo et al. (1995) emphasized the importance of healthy city programs’ institutional indicators as an evaluative tool [24]. Kegler et al. (2000) established evaluation criteria involving structural changes to promote health and health-related public policies [5]. Ahn et al. (2012) included city land use, transportation, and community environment as indicator variables of the DSR (driving force–state–response) model [2]. A healthy living environment includes activities such as restoration of ecological rivers, improvement of fine dust concentration, active bicycle use, water quality inspection visit services, and healthy town developments. The increase in environmentally friendly cars reduces air pollutant emissions, thus, making the urban environment healthier. Facilities and projects related to healthy cities include the expansion of medical institutions and sports facilities where exercise programs, mental health programs, and smoking cessation clinics can be operated. Local governments actively participate in healthy city programs. Healthy city programs are becoming more active as local governments become more interested in the health promotion of local residents. Therefore, the increase of municipal interest in health care increases the effectiveness of healthy city programs. The provision of health-related services by a local body improves the health of local residents. This can be considered as a result of the interest of local governments in promoting the health of local residents. An increase in health-related programs means an increase in initiatives such as healthy urban environment projects, healthy living space programs, healthy living practices, and health services. Therefore, such initiatives can be used as parameters for measuring the performance of healthy city programs. Therefore, the outcomes of healthy city programs have been evaluated based on development of a health-promoting environment, increase in number of eco-friendly vehicles and extent of healthy city facilities, municipality interest in health, health-related municipal services, and healthy city-related local government programs.

The personal happiness index parameter was developed using both psychological and socioeconomic variables. To subjectively measure happiness and diagnose psychiatric diseases such as depression or mania, the classification used in study by Lyubomirsky et al. (1999) was modified and Oxford Happiness Questionnaire (OHQ) was developed. Of the psychological variables, stability, health status, interpersonal relations, and residence with family were adopted. These tools were modified to fit the local society’s healthy cities [25,26]. The United Nations Development Program (UNDP), as used by Schimmel (2007), was also adopted in this study [27]. Personal finances, occupation, and residence are assumed to be correlated with happiness; in the case of finances, happiness or dissatisfaction arise when basic desires are or are not met. Subjective health has been reported as more important than objective health in increasing happiness. Employment status is closely correlated with personal income, job satisfaction leads to happiness, and happier people demonstrate superior work capability. Given this, economic stability, job satisfaction, and health status were incorporated into the measurement [27].

The dependent variable of emotion was evaluated using a depression evaluation survey developed by William et al. (1965). This self-evaluation survey for depression comprises items based on patients diagnosed with depression [28]. Depressed mood, despair, fatigue, and loss of appetite, as measured in the Center for Epidemiologic Studies Depression Scale (CES-D) developed by Radloff (1977), were also adopted [29]. Emotional effects were counter coded such that negativity was calculated as positivity. All variables and items measured are described in Table 1.

### 2.3. Data Collection

The criteria for selection of survey sites were as follows: First, the selection was based on cities that were affiliated with the Korea Healthy Cities Partnership (KHCP) among the local governments promoting healthy city programs. Second, we conducted a survey on the local governments whose healthy city programs were associated with the KHCP. Healthy city programs in Korea are focused on health promotion programs and healthy living environments. Therefore, based on these criteria, we selected the local governments that were actively conducting healthy city programs. Third, in order to compare the results of regional healthy city programs, we divided the cities into metropolitan, independent, and countryside cities. A metropolitan city is a city that is the center of politics, economy, and culture. It is considered to have a population of over 500,000 people. An independent city is a city that integrates cities and counties and refers to cities with a population of over 50,000 people. Countryside cities refer to the eup and myeon areas. The specific analysis targets for metropolitan cities was Seoul; for independent cities, Yongin City, Gyeonggi-do, Changwon, Gyeongsangnam-do; for countryside cities were Yangpyeong County, Gyeonggi-do, Jincheon County, Chungcheongbuk-do, and Buyeo County, Chungcheongnam-do. 

In Seoul, 22 out of 25 boroughs have healthy city programs. In addition, they are centered on businesses such as healthy town and school developments. Therefore, we selected Seoul, which as compared to other metropolitan cities has the most active healthy city programs. In Yongin City, Gyeonggi-do, there are active discussions with citizens on the development of policies related to healthy cities that citizens can positively participate in. Changwon, Gyeongsangnam-do, was selected as an example of favorable health policies in 2016. In Changwon City, a healthy city program is being implemented through citizen-centered living environment creation. The representative healthy city program in Yangpyeong County, Gyeonggi-do is a physical fitness promotion campaign. To this end, the physical education facilities have been expanded to provide a place for citizens to enjoy sports. The improvement of living conditions and the provision of services for the community will address urban inequality. In addition, social infrastructure will facilitate the provision of health services. In fact, Yangpyeong County increased its population by 30.99% through the revitalization of health businesses including the establishment of healing health support centers [32]. In the case of Jincheon-gun, Chungcheongbuk-do, a healthy city homepage has been created to provide health-related services to revitalize healthy city businesses. In addition, the healthy city program that is being visited is creating an atmosphere for practicing healthy living for the local residents. Buyeo County, Chungcheongnam-do is implementing a program to improve the health behaviors of local residents through the operation of a happy health village.

On- and off-line surveys were conducted for local residents in selected areas. The selected subjects were public officials in charge of healthy city programs, related organizations, medical facility workers, and local residents. For diversity of the sample, the experts of the healthy cities were included and the selection criteria included those living in the healthy city program areas. The survey targets were appropriately selected according to age, sex, and residential areas. There are many healthy city programs in Korea. Among them, a survey was conducted on local residents who were part of health promotion programs dealing with issues such as obesity, smoking, and improving living habits, and projects such as creation of villages and parks as part of healthy living areas. Offline surveys were conducted in metropolitan cities from 25 September to 10 October 2017. A total of 200 questionnaires were distributed and 196 (98.0%) questionnaires were collected. In the independent and countryside cities, online surveys were conducted from 14 to 17 August, 2018. Each of the 250 questionnaires were distributed, and 200 questionnaires were retrieved (80%) from the independent cities and 200 (80%) from the countryside. The survey was conducted focusing on the subjects involved in healthy city programs and a total of 596 questionnaires were collected and used for analysis.

In a previous research design, only Seoul, which has the largest number of local governments participating in the healthy city programs, was selected as the research target. However, the results of the healthy city programs varied depending on the policies of the different local governments of the healthy cities. Therefore, healthy city program performance was compared by region and further analyzed by adding independent and countryside cities to the research targets.

### 2.4. Method

The collected data were analyzed using SPSS 25.0 (SPSS Inc., Chicago, IL, USA) and Amos 18.0 programs (SPSS Inc., Chicago, IL, USA). The analysis method was as follows:

(1) The general characteristics of the survey subjects were calculated as frequency and percentage.
(2) Factor analysis was conducted to verify the validity of healthy city program performance, personal happiness index, and emotional influences, and its reliability was verified.
(3) An independent sample *t*-test was conducted to compare the difference in regional healthy city program performance, individual happiness index, and emotional effects.
(4) A structural model was used to analyze the structural impact of the performance of the healthy city program on emotions through the Amos Program.

## 3. Results

### 3.1. Demographics

The participants’ demographics are shown in Table 2. A total of 596 individuals took part in the study, 248 men and 348 women. The difference in residual expectation of life and healthy life years of Korean males is about 9 years, and about 12 years for females. Women’s residual expectation of life and healthy life years was higher than men’s; however, the period in which a woman can live a healthy life was observed as short. Women are more likely to participate in healthy city projects than men because they want to improve their health status, especially those in the vulnerable groups, and achieve health equity. Therefore, according to the survey, the participants included more women than men. The mean age for all participants was 37.5 years; large cities had a higher mean age, at 41.4 years. A predominant proportion of participants had college or higher education (254, 45.5%). The largest income range proportion was 5 million Won or more (152, 25.5%), followed by 2–3 million Won or less (117, 19.6%). Occupation showed a predominant proportion of office workers (150, 25.2%), followed by students (94, 15.8%) and teachers/specialized jobs (66, 11.1%).

### 3.2. Reliability and Study Model Verification

Cronbach’s *α* was used to confirm the reliability and validity of each variable. A value of 0.6 or higher is regarded as reliable. Table 3 shows the number of items in each variable and their reliabilities. The independent variable, healthy city program performance, was composed of six items. The dependent variable of emotional effects and the personal happiness index parameter were composed of nine items each. Each variable’s *α* values were satisfactory, at 0.6 or higher.

Confirmative factor analysis was used to confirm the study’s validity. One of the six items from healthy city program performance, two of the nine items from personal happiness index, and two of the nine items from emotional effects had mean factor loading values below the cutoff of 0.5. Therefore, these items were excluded, and only the satisfactory items were included in the analysis. Discriminant validity was evaluated by comparing the average variance extracted value and the squared correlation values. Because the squared value of the latent variables’ correlation values was less than the average variance extracted value, discriminant validity was confirmed.

### 3.3. Analysis of Average Difference

In Korea, there is a discrepancy in health levels between different regions. The effect of the city environment on individuals’ health has been discussed as a cause for this. A city’s shape, landscape use, public transportation, and proximity to major facilities significantly affect local residents’ obesity; stress; and walking, cycling, and physical activity habits. It is highly probable that healthy city program performance, personal happiness index, and emotional effects differ according to city type. Therefore, a *t*-test was performed to analyze the differences between metropolitan cities, independent cities, and the countryside.

First, the mean difference between metropolitan cities and the countryside was analyzed. The results showed significant differences between metropolitan cities and the countryside (Table 4). Countryside regions showed higher healthy city program performance, personal happiness index, and emotional effects values than did metropolitan cities. Countryside residents were more health-friendly than those of metropolitan cities, leading to better healthy city program performance. Metropolitan city residents experience less subjective happiness and positive emotion than countryside residents; this is inferred to be a result of lack of community ties and city expansion due to drastic urbanization.

The mean difference between metropolitan and independent cities was then analyzed. The results showed significant differences between metropolitan and independent cities (Table 5). Similar to the countryside, independent cities showed higher healthy city program performance, personal happiness index, and emotional effects values than did metropolitan cities. In metropolitan cities, accessibility to public transportation and residence density limit local residents’ physical activity. Therefore, decreased participation in the healthy city program led to lower performance than was observed in independent cities. Furthermore, the psychological health of metropolitan city residents was worse than that of independent city residents, reflecting low levels of happiness and optimism [33].

### 3.4. Structural Equation Modeling Verification

**Figure 2:**
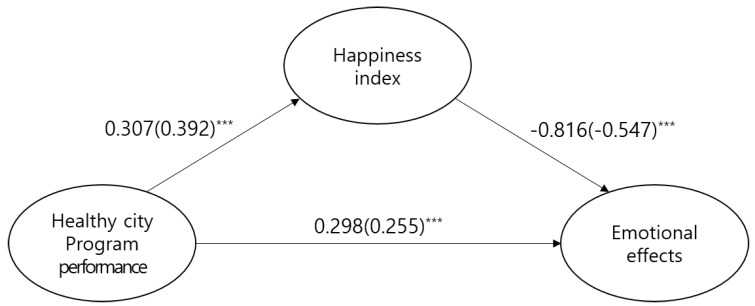
Research model path coefficients. Note: *** *p* < 0.001.

Results of analysis of the model in this study are shown in Table 6. The indices satisfied the general fit. The study model’s fitness and path coefficients are as follows (Table 6 and Table 7, Figure 2).

The path coefficients of each study model showed that emotions have significant static effects on healthy city program performance (*β* = 0.298, C.R. = 6.966, *p* < 0.001). In contrast, personal happiness index showed significant negative effects on emotions (*β* = −0.816, C.R. = −7.868, *p* < 0.001). 

To determine the significance of indirect effects, the bootstrapping method was used. Healthy city program performance was observed to have significant indirect effects on emotions using personal happiness index as a parameter (*B* = −0.250, *p* = 0.009). Therefore, personal happiness index acts as partial mediator between healthy city program performance and emotions.

## 4. Discussion

This study investigated the structural effects of healthy city program performance on local residents’ emotions and the personal happiness index parameter. The subjects of the study were residents of metropolitan cities, independent cities, and countryside regions participating in healthy city programs. Metropolitan city residents were selected from Seoul, as local autonomous districts in Seoul are actively conducting healthy city programs.

First, we compared the mean static effects of healthy city program performance, personal happiness index, and emotional effects in different regions. The results showed that healthy city program performance, personal happiness index, and emotional effects were all higher in countryside regions and independent cities than in metropolitan cities. In metropolitan cities, highly developed transportation results in limited walking in daily life. Additionally, a crowded environment results in increased stress and irregular eating habits, leading to decreased physical activity and unhealthy diet. Metropolitan cities thus demonstrated relatively lower healthy city program performance [34,35]. In this study, personal happiness index was measured according to satisfaction with health, family life, interpersonal relationships, and daily life security. The happiness index was higher in countryside regions than metropolitan cities, consistent with the results of previous studies [36,37,38]. These results are due to the fact that more time is spent with family and in local community activities in the countryside than in metropolitan cities [22]. Residents of metropolitan cities are additionally exposed to environmental hazards such as micro-dusts, crime, and high stress perception. They are thus less emotionally stable than residents of the countryside and independent cities, leading to relatively lower emotional effect averages. The physical environment of the city affects the health of the residents. Looking at this environment and the health of the local residents as a driving force–state–response (DSR) model, it has been shown that water quality, air quality, and crime affect the health of local residents. Especially in metropolitan cities, the health of the population is threatening the health and stability of cities. Therefore, if healthy city programs are extended to metropolitan areas, they will improve the psychological stability of the residents in the urban areas [39].

Structural equations were used to analyze the structural effects and correlation between healthy city program performance, personal happiness index, and emotions. The results showed that healthy city program performance and emotions were significant when personal happiness index was used as a parameter. Specifically, healthy city program performance had significant static effects on emotion. In other words, the results of the healthy city programs seem to have positively influenced the emotions of local residents. Healthy cities want to alleviate their physical, social, and emotional problems through health promotion activities rather than treating them. Therefore, the results of the performance of healthy city programs seem to have stabilized the emotions of local residents. This shows that healthy city programs decrease local residents’ negative emotions, such as stress and depression [35]. Appropriate emotional responses to change in the environment indicates adaptation to the environment, and emotional stability indicates a healthy psychological state. In other words, healthy city programs, which decrease negative health factors, have positive effects on individuals’ emotions [19]. Therefore, healthy city programs stabilize residents’ emotions by increasing health friendliness.

In addition, as healthy city program performance improve, personal happiness index increases. This result coincides with a previous study’s results reporting that subjective welfare affects life satisfaction and improves personal happiness index [40]. Happiness encompasses life satisfaction, positive emotions, and negative emotions. Individuals that perceive high levels of positive emotion are seen as happy. Happy individuals perceive their own health well and actively engage in activities to improve their health, ultimately improving their objective physical health [7].

Finally, the parameter effects of personal happiness index on healthy city program performance and emotions were investigated. Healthy city program performance had negative effects on emotion using personal happiness index as a parameter. The sources and definition of happiness and motivation differ based on individuals’ daily life experiences. Financial security, good family relationships, and social relationships are thought to bring happiness to an individual. However, this is not the case for all. Some individuals feel happiness in response to a comfortable life, whereas others do not. Furthermore, individuals can feel happiness and pride in one month and despair in the next. These observations are due to the fact that subjective happiness differs greatly based on setting and time [25]. Happiness level also varies in different regions. Life satisfaction and capital assets are closely correlated. Policies that regulate local society also correlate with happiness. Strong policies on healthy cities make life safer and more predictable, increasing happiness [41]. Happiness involves positive and negative emotions, and a mixture of happiness and sorrow. Happiness seems to be the opposite of sorrow; however, there can also be mixed feelings of happiness and sadness at the same time. Larsen (2001) states that people are stressed in comfort, happiness, and sadness. Those who are satisfied with the present conditions are happy, but at the same time feel sad. Therefore, there seems to be a negative relationship between the personal happiness index and emotions because people have mixed emotions and it is difficult to separate them [42]. Therefore, healthy city program performance has positive effects on personal happiness, and happiness is normally closely correlated with emotions. However, individuals perceive happiness differently, and happiness changes over time. Thus, the personal happiness index has negative effects on emotions.

## 5. Conclusions

This study confirmed the structural correlation between healthy city program performance, personal happiness index, and emotions. The following efforts are necessary to improve healthy city program performance. First, healthy city programs should be designed according to the region’s size and environment. Emphasis should be placed on healthy city activities centered in residence areas and welfare facilities and services based on the city’s size. The goal of a healthy city program is to allow residents to carry out every aspect of their lives. Health promotion also improves physical, psychological, and social health by preventing, treating, and rehabilitating disease [18]. Health-promoting healthy city programs should continuously improve residents’ living environment to bring stability to their daily lives. To this end, use of health complex facilities and programs allowing communication with residents should be further enhanced. Physical infrastructure and refreshing natural environments could improve residents’ subjective health satisfaction, and improved communication with residents could provide meaning in life by improving social activity.

Second, healthy city programs should be continuously managed through policy amplification. Currently, many local governments conduct healthy city programs. However, passive operation is observed in many cases. A healthy city program is a realization of political will, a long-term project that coexists with the city. Short-term healthy city program planning should be avoided [2]. In Korea, the direction of healthy city programs are frequently altered when the head of local government changes. Whenever new funding is acquired, new programs are started and the previous programs lose structure. To induce formation of local community and improve quality of life, it is necessary to make policies through communication. This would improve the participation of local society and lead to long-term development of the city environment [42]. Therefore, local governments that conduct healthy city programs should consider them a long-term project and should maintain a coherent attitude to qualitatively enhance them. Simultaneously conducting healthy city programs and health promotion programs is also effective at the central government level. A healthy city program reference manual should be distributed to promote sustainable programs.

Third, continuous and systematic monitoring of healthy city programs is necessary. Currently, most local governments lack effective program development and management. Haphazard program development results in unnecessary programs or discontinuation of activities. Additionally, because healthy cities require cooperation between city components, different departments are connected by a network. Healthy city teams have been formed, but these participate only in health promotion departments in welfare centers. Based on the healthy city program’s characteristics, the welfare center should act as the center of the program. However, welfare centers lack authority in Korea, and cannot effectively lead healthy city programs. The promotion of health programs in Korea started in 1999 as a demonstration of health-promotion-based welfare centers in 12 places. Subsequently, in 2002, this was expanded into 100 welfare centers covering 4 areas which include smoking and drinking cessation, exercise, nutrition, and health practices, and now health promotion programs are being carried out in public welfare centers across the country. Healthy cities consist of area-based health promotion programs that are part of the general health care policy. The implementation of healthy city programs may overlap with the policies implemented by public welfare centers. If a healthy city program is conducted centering on public welfare centers, ongoing monitoring can be achieved. Welfare center-based healthy city programs should be developed and monitored using a network of different departments. This would be helpful in determining health-promoting variables in local residents and would allow for effective management of healthy city programs. The limitations of this study are as follows. First, we failed to analyze the healthy city programs’ performance over time. Healthy city programs are divided into short, medium, and long term depending on the time limits of the program. Therefore, in future research, it is necessary to analyze the effects of the results of the healthy city programs on the local residents by dividing the results into short, medium, and long term. Second, there is no comparative analysis between the regions where healthy city programs are implemented and those where they are not. If a healthy city program has a positive effect on the local residents’ emotions, there will be a difference in the emotions of the residents in the areas where healthy city programs are not implemented. Therefore, it is necessary to analyze the influence of the healthy city programs through analyzing the emotional effects and individual happiness index of the residents in the areas where healthy city programs are enforced and areas where such programs are not implemented.

## Figures and Tables

**Table 1:** Composition of the survey.

Dimension	Variable	Item	Reference
Independent variable	Healthy city program performance	Development of health-promoting environment Increase in number of eco-friendly vehicles Extent of healthy city facilities and projects Municipality interest in health Health-related municipal services Increase in healthy city-related programs	[2,5,24]
Parameter	Personal happiness index	Increase in psychological stability Residence with a partner or family Good interpersonal relations Adaptation to local society Joy and happiness in daily life Financial stability Job satisfaction Health status Residence status	[25,26,27,30]
Dependent variables	Emotional effects	Physical discomfort Fatigue Anger or irritability Nervousness Health concerns Depression Appetite Sad thoughts	[18,28,31]

**Table 2:** Participants’ demographic characteristics (*n* = 596).

Characteristic	Type	Metropolitan City		Independent City		Countryside		Total	
		*n*	%	*n*	%	*n*	%	*n*	%
Gender	Male	100	51.0	82	41.0	66	33.0	248	41.6
	Female	96	49.0	118	59.0	134	67.0	348	58.4
Age	Mean age	41.4		36.1		35.0		37.5	
Education	No formal	1	0.5	0	0	0	0	1	0.2
	Elementary school	3	1.5	2	1.0	3	1.5	8	1.3
	Middle school	8	4.1	8	4.0	11	5.5	27	4.5
	High school	84	42.9	53	26.5	46	23.0	183	30.7
	Junior college	36	18.4	38	19.0	32	16.0	106	17.8
	University or above	64	32.6	99	49.5	108	54.0	254	45.5
Income level	Under 1 million won	16	8.2	7	3.5	5	2.5	28	4.7
	1–2 million won	32	16.3	22	11.0	26	13.0	80	13.1
	2–3 million won	47	24.0	36	18.0	34	17.0	117	19.6
	3–4 million won	29	14.8	38	19.0	43	21.5	110	18.5
	4–5 million won	35	17.9	38	19.0	36	18.0	109	18.3
	5 million won and over	37	18.9	59	29.5	56	28.0	152	25.5
Occupation	Agriculture, Stock breeding	1	0.5	1	0.5	1	0.5	3	0.5
	Self-employed	30	15.3	8	4.0	12	6.0	50	8.4
	Office clerk	4	2.0	80	40.0	66	33.0	150	25.2
	Teacher, Professional	38	19.4	13	6.5	15	7.5	66	11.1
	Homemaker	6	3.1	27	13.5	23	11.5	56	9.4
	Civil servant	20	10.2	4	2.0	6	3.0	30	5.0
	Student	36	18.4	26	13.0	32	16.0	94	15.8
	Service	33	16.8	8	4.0	15	7.5	56	9.4
	Manufacturing	2	1.0	10	5.0	13	6.5	25	4.2
	Unemployed	7	3.6	8	4.0	10	5.0	25	4.2
	Other	19	9.7	15	7.5	7	3.5	41	6.9

**Table 3:** Reliability verification.

Variable	Items	Construct Reliability (Cronbach’s *α*)
Healthy city program performance	6	0.846
Personal happiness index	9	0.793
Emotional effects	9	0.837

**Table 4:** Analysis of average differences between metropolitan cities and countryside.

Factor	Metropolitan Cities	Countryside	*t*-Value
Healthy city program performance	3.01 (0.702)	3.21 (0.587)	−3.111 **
Personal happiness index	3.03 (0.329)	3.12 (0.305)	−2.792 **
Emotional effects	2.83 (0.598)	3.22 (0.633)	−6.256 ***

** *p* < 0.05, *** *p* < 0.001.

**Table 5:** Analysis of average differences between metropolitan cities and independent cities.

Factor	Metropolitan Cities	Independent Cities	*t*-Value
Healthy city program performance	3.01 (0.702)	3.28 (0.621)	−4.040 ***
Personal happiness index	3.03 (0.329)	3.12 (0.299)	−3.063 **
Emotional effects	2.83 (0.598)	3.30 (0.648)	−7.369 ***

** *p* < 0.05, *** *p* < 0.001.

**Table 6:** Research model verification.

Model	*X* ^2^	*DF*	*p*-Value	GFI	TLI	CFI	RMR
Research model	371.224	147	0.001	0.94	0.94	0.95	0.04

*X*^2^ = Chi-square statistic, *DF* = Degrees of freedom, GFI = Goodness of fit index, TLI = Tucker Lewis index, CFI = Comparative fit index, RMR = Root mean square residual.

**Table 7:** Research model path coefficients.

Path	*B*	*β*	S.E.	C.R.
Program performance → Happiness	0.307	0.392	0.044	6.966 ***
Happiness → Emotional effects	−0.816	−0.547	0.104	−7.868 ***
Program performance → Emotional effects	0.298	0.255	0.061	4.854 ***

*** *p* < 0.001, *B* = Unstandardized coefficients, *β* = Standardized coefficients, S.E. = Standard error, C.R. = Critical ratio.
